# Numerical Study to Analyze the Influence of Process Parameters on Temperature and Stress Field in Powder Bed Fusion of Ti-6Al-4V

**DOI:** 10.3390/ma18020368

**Published:** 2025-01-15

**Authors:** Helia Mohammadkamal, Fabrizia Caiazzo

**Affiliations:** Department of Industrial Engineering, University of Salerno, 84084 Fisciano, SA, Italy; f.caiazzo@unisa.it

**Keywords:** additive manufacturing, laser powder bed fusion, Ti-6Al-4V alloy, finite element method, thermo-mechanical modeling, thermal field, melt pool, thermal residual stress

## Abstract

This paper presents a comprehensive numerical investigation to simulate heat transfer and residual stress formation of Ti-6Al-4V alloy during the Laser Powder Bed Fusion process, using a finite element model (FEM). The FEM was developed with a focus on the effects of key process parameters, including laser scanning velocity, laser power, hatch space, and scanning pattern in single-layer scanning. The model was validated against experimental data, demonstrating good agreement in terms of temperature profiles and melt pool dimensions. The study elucidates the significant impact of process parameters on thermal gradients, melt pool characteristics, and residual stress distribution. An increase in laser velocity, from 600 mm/s to 1500 mm/s, resulted in a smaller melt pool area and faster cooling rate. Similarly, the magnitude of residual stress initially decreased and subsequently increased with increasing laser velocity. Higher laser power led to an increase in melt pool size, maximum temperature, and thermal residual stress. Hatch spacing also exhibited an inverse relationship with thermal gradient and residual stress, as maximum residual stress decreased by about 30% by increasing the hatch space from 25 µm to 75 µm. The laser scanning pattern also influenced the thermal gradient and residual stress distribution after the cooling stage.

## 1. Introduction

Additive manufacturing (AM) has revolutionized the landscape of modern manufacturing, offering unparalleled design freedom, reduced lead times, and the ability to produce highly complex components with intricate geometries [1,2]. Among the various AM techniques, Laser Powder Bed Fusion (LPBF), also known as Selective Laser Melting (SLM), has special importance for its ability to fabricate metal parts layer by layer, directly from three-dimensional (3D) digital models [3]. LPBF involves the precise deposition of powder layers onto a substrate [4,5] followed by selective melting using a high-power laser beam. This process enables the creation of parts with exceptional mechanical properties [3,6,7], making it particularly attractive for applications in aerospace [8], automotive [9], and medical industries [10].

However, achieving optimal properties and performance in Ti-6Al-4V parts fabricated via LPBF relies heavily on understanding and controlling the thermal behavior during the process [11,12]. The thermal dynamics in LPBF are inherently complex, governed by intricate interactions between the laser beam, powder bed, and surrounding environment. As the laser beam selectively melts the powder layers, it generates localized heating, leading to rapid solidification and thermal gradients within the material. These thermal cycles profoundly influence various aspects of the final part, including microstructural evolution [12,13,14,15], mechanical properties [16,17], and residual stress formation [7,18,19,20]. Thermal stress can result in deformation and distortion, negatively impacting the dimensional precision of the final product. If not properly controlled, this stress can even cause the failure of the object during production [21,22]. Therefore, controlling the thermal gradient and thermal stress is crucial throughout the manufacturing process. Optimization of process parameters, including laser velocity, laser power, powder layer thickness, hatch space, and scanning pattern, is the main key to minimizing stress formation and controlling the thermal gradient by reducing the cooling rate [19,23,24,25].

Developing a reliable thermomechanical simulation model is beneficial for optimization of process parameters. It can minimize the need for experimental processes and save expenses [26]. A validated thermo-mechanical model is crucial in understanding the thermal and mechanical behavior during the LPBF process. It allows for the prediction of thermal history and accurate forecasting of thermal stress formation. This approach is essential for analyzing how individual process parameters, such as laser power and velocity, influence temperature distribution and stress development, ultimately enabling the optimization of these parameters to minimize thermal residual stresses [27,28].

Much research has been focused on developing a thermo-mechanical model to study the LPBF process and the role of process parameters. For instance, Mishra et al. used a simulation model to study the effect of laser power and velocity on melt pool dimension at constant energy density during the LPBF process [29]. Another modeling investigation on melt pool dimensions was performed by E. Soylemez, using a volumetric double ellipsoid heat source [30]. Chen et al. focused on the role of laser jump speed on temperature history and stress distribution by developing a thermo-mechanical finite element model [28]. Hussein et al. simulated the LPBF process to predict the temperature and residual stress distribution for the single-layer process [31]. They also found the maximum stress location along the laser scanning. Xioa et al. investigated the role of different process parameters, including laser velocity, power, and hatch space, on residual stress using 3D FEM. They concluded that there is a complex relationship between these process parameters and thermal residual stress that ended with no monotonous changes [18]. The role of other process parameters such as ambient temperature and substrate thickness was also investigated in a numerical study carried out by Liu et al. [32]. The overlap between the lines was also another process parameter that was studied by Chen et al. [33]. They used a simulation model to study the role of overlap rate on final residual stress. A finite element analysis was performed by Luo et al. to predict the temperature and stress field in the LPBF process [34]. Parry et al. developed a thermo-mechanical simulation, which used a volumetric heat source based on the Goldak model, to compare two different scan strategies [35]. A multi-layer simulation model was also developed by Ganeriwala et al. to investigate stress formation during the LPBF process [36]. Li et al. also introduced a thermomechanical model for the multi-layer LPBF process in order to study the effect of residual stress on final fabricated parts [37].

As previously noted, many thermomechanical models have been developed to simulate the LPBF process. However, numerous factors can impact the reliability of the simulation outcomes, with even minor changes in input parameters potentially altering the final results. Key aspects such as the governing physics, assumptions made, heat source modeling, and initial boundary conditions can significantly influence the results [38,39,40]. Consequently, ongoing efforts to improve simulation models are crucial for achieving more accurate and trustworthy research outcomes. Zhang et al. showed that by considering absorptivity as a function of velocity and power, the predictions of melt pool dimensions could improve significantly [41]. Similarly, Mohammadkamal et al. concluded that a FEM using a volumetric heat source model with absorptivity decaying function ended in more reliable outcomes compared to other heat source models [42]. However, to date, no model has simultaneously predicted thermal gradients and residual stress distribution while integrating both modified thermal absorptivity and a volumetric heat source with an absorptivity decaying function, as evidenced by the limited scope of existing studies [26,28,30,31,34,43,44]. Additionally, a significant scientific gap remains in track-scale thermo-mechanical modeling, as most prior studies have focused on multi-layer models. The limitation of multi-layer models lies in their inability to predict defects that may develop within intermediate layers. In contrast, track-scale studies enable the investigation of thermal stress evolution and defect formation with greater precision.

In this study, the impact of key process parameters, including laser velocity, laser power, hatch space, and scanning pattern, on the temperature and stress fields was analyzed. This was carried out using a three-dimensional volumetric heat source with an absorptivity decaying function, where absorptivity is modeled as a function of both laser power and velocity.

## 2. Materials and Methods

This section outlines the materials and methods used to simulate and experimentally validate the LPBF process for Ti-6Al-4V. It begins by describing the material properties and modeling assumptions, followed by the governing equations and computational setup, and concludes with the experimental procedures employed for model validation. Together, these subsections provide a comprehensive framework for understanding the study’s methodological approach.

### 2.1. Materials

In the present study, Ti-6Al-4V is used as a powder layer and solid substrate, in both the simulation model and experiments. To achieve more accurate results, all thermal and mechanical properties in the model are considered temperature-dependent functions. This approach enables the model to more precisely represent the actual behavior of material under varying temperature conditions. Table 1 and Table 2 present the thermal and mechanical properties of Ti-6Al-4V used for simulation modeling.

### 2.2. Modeling Methodology

#### 2.2.1. Model Assumptions

Due to the complexity of the LPBF process, and numerous effective factors, the following assumptions are made to simplify the FEM: (a) The simulation model is developed in conduction mode only, and fluid flow is not considered. This choice is made to reduce computational complexity and focus on the thermomechanical aspects of the process. (b) The evaporation of material is neglected during the simulation to keep the model simpler and reduce computational demands; however, the parts of material which experience temperature over the $T_{v}$ is considered a vapored part in the results and discussion section. This approach ensures that the model remains efficient while still addressing the key thermal effects related to evaporation. (c) The powder layer is considered a porous material, with an initial porosity of 0.35, and discriminated from solid substrate by different density and heat conductivity according to the following equations [30]:

(1) $$\frac{\rho_{P}}{\rho_{S}}=1-\varphi$$

(2) $$\frac{k_{p}}{k_{s}}=\frac{1-\varphi }{1+11\varphi^{2}}$$

In Equations (1) and (2), $\rho_{P}$, $\rho_{S}$, $k_{p}$, and $k_{s}$ are the density and thermal conductivity of powder and solid material, respectively. The initial porosity in the powder bed is indicated as φ in those equations. This assumption is widely used in LPBF simulations [30,31,47] because it provides a practical way to account for the thermal and physical differences between the powder and solid material. (d) Finally, in the analysis of thermal stress and solid mechanics, the influence of powder bed is neglected due to the insignificant effect of powder particles.

In this study, the FEM approach was selected over CFD to prioritize computational efficiency and maintain focus on the thermomechanical aspects of the problem. While the neglect of fluid flow typically results in the underestimation of melt pool dimensions [29], this effect is addressed by incorporating other considerations, such as a suitable heat source model and the modification of thermal absorptivity [43], which help to compensate for the simplifications made. These considerations ensure reliable predictions of the melt pool shape while minimizing computational costs.

#### 2.2.2. Governing Equations

##### Thermal Analysis

For the 3D thermal model, the governing heat transfer equation can be expressed as follows:

(3) $$\rho c_{p}\frac{\partial T}{\partial t}=\frac{\partial }{\partial x}\left(k\frac{\partial T}{\partial x}\right)+\frac{\partial }{\partial y}\left(k\frac{\partial T}{\partial y}\right)+\frac{\partial }{\partial z}\left(k\frac{\partial T}{\partial z}\right)+\bar{Q}-\rho L\frac{\partial fs}{\partial t}$$

This equation is derived from the general heat transfer equation, incorporating an additional term to account for the release or absorption of latent heat during phase changes. Where ρ, *c_p_*, *k* and *L* represent the density, specific heat capacity, thermal conductivity, and latent heat of the material, respectively. The term f_s_ is the solid fraction which varied from 0 (fully liquid) to 1 (fully solid), according to the following equation:

(4) $$f_{s}=\left\{\begin{array}{ll} 0 & T<T_{S}\\ \frac{T_{L}-T}{T_{L}-T_{S}} & T_{S}⩽T⩽T_{L}\\ 1 & T>T_{L} \end{array}\right.$$

Finally, the resulting equation is as follows:

(5) $$\rho \left(c_{p}-\frac{L}{T_{L}-T_{S}}\right)\frac{\partial T}{\partial t}=\frac{\partial }{\partial x}\left(k\frac{\partial T}{\partial x}\right)+\frac{\partial }{\partial y}\left(k\frac{\partial T}{\partial y}\right)+\frac{\partial }{\partial z}\left(k\frac{\partial T}{\partial z}\right)+\bar{Q}$$

*x*, *y* and *z* are the coordinate directions in a Cartesian system. $\bar{Q}$ is also a symbol for the volumetric heat, generated by laser (W/m^3^) during the process to heat up and melt the material. For simulating the process in this study, a moving heat source is defined based on the volumetric Gaussian heat source model, with absorptivity decaying function which can be described as below:

(6) $$\begin{array}{c} I(x,y,z)=\frac{2P}{\pi r^{2}}\exp\left[-2\frac{{\left(x-(Vt-x_{0})\right)}^{2}+y^{2}}{r^{2}}\right]\cdot \frac{\beta }{th}\cdot \exp\left[-\frac{\left|z\right|}{th}\right] \end{array}$$

where P is the laser power (W), V is the laser velocity (mm/s), r is the laser radius (µm) and th is the laser penetration depth which is considered powder layer thickness (µm). In Equation (6), β is the laser absorptivity and, in this study, it is considered the function of laser power (P) and velocity (V) based on previous research findings [44] as described in the following equation:

(7) $$\beta (P,V)=a_{1}\frac{P}{\sqrt{V}}+b_{1}$$

where $a_{1}$ and $b_{1}$ are constant and equal to 0.036 and 0.48, respectively. Moreover, for more accurate results, thermal convection and radiation are also considered boundary conditions on the top surface to calculate heat loss flux. The convective heat exchange between the top surface and the surrounding area can be calculated as follows:

(8) $$q_{conv}=h\left(T-T_{0}\right)$$

where h is the coefficient of heat convection for Ti-6Al-4V and equal to 10–25 W/m^2^·K (depending on the temperature) and *T*_0_ is the ambient temperature. The surface radiation heat loss equation can also be stated as below:

(9) $$q_{rad}=\varepsilon \sigma_{SB}\left(T^{4}-T_{0}^{4}\right)$$

where $\sigma_{SB}$ is the Stephen–Boltzmann constant equal to 5.67 × 10^−8^ W/m^2^·K^4^ and ε is the emissivity.

##### Stress Analysis

The structural behavior of the system is governed by the following fundamental equations, which account for the relationship between stress, strain, and elastic–plastic hardening model:

(10) $$\{\sigma \}=[D]\left\{\varepsilon^{e}\right\}$$

(11) $$\left\{\varepsilon \right\}=\left\{\varepsilon^{e}\right\}+\left\{\varepsilon^{p}\right\}+\left\{\varepsilon^{t}\right\}$$

where σ is the stress vector, D is the elasticity matrix, and $\varepsilon^{e}$, $\varepsilon^{p}$, and $\varepsilon^{t}$ are elastic, plastic and thermal strain, respectively. Thermal strain is defined as follows:

(12) $$\varepsilon^{t}=\int_{T_{ref}}^{T}\alpha_{e}(T)dT$$

where $\alpha_{e}$ is the coefficient of thermal expansion.

Since in structural study, the material is considered to be isotropic; strain and stress relationship along different directions of Cartesian coordinates can be written as follows:

(13) $$\varepsilon_{x}=\frac{\sigma_{x}-\mu \left(\sigma_{y}+\sigma_{z}\right)}{E}+\varepsilon_{x}^{p}+\varepsilon^{t}$$

(14) $$\varepsilon_{y}=\frac{\sigma_{y}-\mu \left(\sigma_{z}+\sigma_{x}\right)}{E}+\varepsilon_{y}^{p}+\varepsilon^{t}$$

(15) $$\varepsilon_{z}=\frac{\sigma_{z}-\mu \left(\sigma_{y}+\sigma_{x}\right)}{E}+\varepsilon_{z}^{p}+\varepsilon^{t}$$

where μ represents the Poisson’s ratio and E signifies elastic modulus. Plasticity was modeled using the Swift model, which is represented by the following equation:

(16) $$\sigma_{ys}=\sigma_{ys0}{\left(1+\frac{\varepsilon_{p}}{\varepsilon_{0}}\right)}^{n}$$

where $\sigma_{ys}$ and $\varepsilon_{0}$ are the yield strength and reference strain, respectively. The hardening exponent is represented by *n* in the equation which varies from 0.3 at room temperature to 0.01 near melting point.

Residual stress is a key factor in the LPBF process, influencing mechanical performance, dimensional accuracy, and stability of the components. Its measurement helps in understanding the thermomechanical behavior during and after the process, aiding in the optimization of process parameters for improved quality and reliability [20,48,49,50].

In order to measure the residual stress, von Mises stress is also used as a common criterion to predict the yielding of materials:

(17) $$\sigma_{e}=\sqrt{\frac{1}{2}\left[{\left(\sigma_{x}-\sigma_{y}\right)}^{2}+{\left(\sigma_{y}-\sigma_{z}\right)}^{2}+{\left(\sigma_{z}-\sigma_{x}\right)}^{2}+6\left(\tau_{xy}^{2}+\tau_{yz}^{2}+\tau_{zx}^{2}\right)\right]}$$

In Equation (17), $\tau_{xy}$, $\tau_{yz}$, and $\tau_{zx}$ represent the shear stresses in the *xy*, *yz*, and *zx* planes, respectively.

#### 2.2.3. Model Dimensions, Meshing, and Time Steps

**Figure 1:**
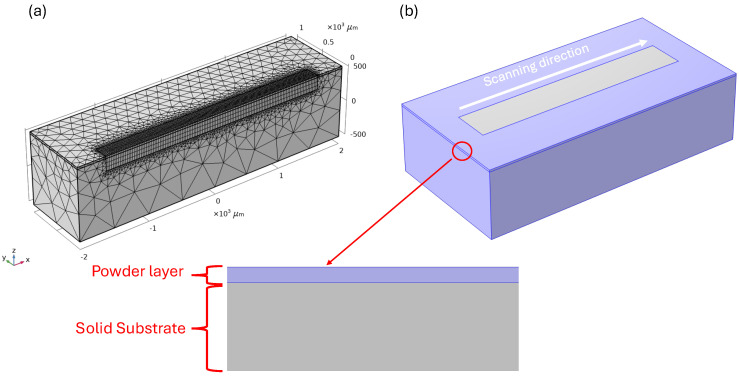
Schematic of model for (**a**) single-track and (**b**) multi-track studies.

In the present work, the thermo-mechanical models were developed using FEM-based simulation software, COMSOL Multiphysics version 5.6, to study the thermal history and structural behavior during and after laser scanning. Two rectangular models were designed to optimize computational time: one with dimensions of 4000 μm × 1000 μm × 1000 µm (Figure 1a) for single-track studies, and another with dimensions of 4000 μm × 2000 μm × 1000 µm (Figure 1b) for multi-track studies. In the single-track model, symmetrical division helps to further optimize the computational efficiency by reducing the size of the model that needs to be processed. A 30 μm layer of powder was applied directly on the surface of the solid substrate, as described in Section 2.2.1. The laser scanning direction is along the x-axis, the y-axis is perpendicular to the laser scanning direction, and the z-axis is vertical, pointing upward relative to the x-axis.

Tetrahedral mesh elements were used in the models, with finer elements in the scanning area (approximately 40 μm) that gradually became coarser farther from the scan. The total number of elements was 17,442 for halved design and 45,255 for complete geometry. During the laser scanning process, a time step was around 1 µs, while for the cooling stage, a time step of 25 µs was used. The initial temperature and atmosphere for all simulation approaches were set to 308 K and 0.1% O_2_, respectively.

### 2.3. Experimental Procedure

**Figure 2:**
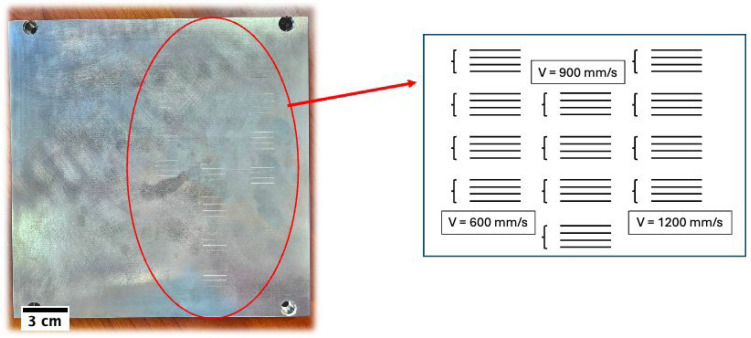
Experimental sample and detailed pattern.

To validate the simulation model with experiments, alongside the data collected from previous experimental articles [51,52], additional tests were also conducted using the EOSINT M 270 LPBF machine (EOS GmbH, Krailing, Germany). The experimental setup followed standard procedures widely adopted in the additive manufacturing field for Laser Powder Bed Fusion (LPBF) processes [51,52,53,54]. Ti-6Al-4V material was used for both the build plate and feedstock powder to mitigate any distortion and deformation arising from differences in elongation or expansion coefficients. The powder particles were spherical with an average size of 30 µm. The tests began with an initial temperature of 308 K and 0.1% of O_2_ inside the chamber. The process parameters were set with a laser power of 170 W and three different laser velocities of 600, 900, and 1200 mm/s. Each test was repeated under constant conditions 16 times to assess the repeatability of the experimental results. Figure 2 provides a detailed view of the experimentally deposited lines on the build plate.

## 3. Results and Discussion

### 3.1. Model Validation

**Figure 3:**
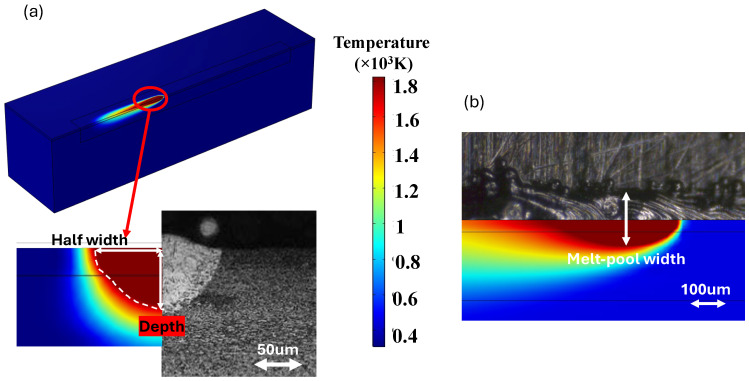
(**a**) Melt pool dimensions including depth and width, obtained from simulation with a laser power of 150 W and a velocity of 1200 mm/s, compared with the experiment conducted by Dilip et al. [52]; and (**b**) melt pool width from the simulation with a laser power of 170 W and a velocity of 600 mm/s, compared to the experiments described in Section 2.3.

To validate the simulation model, a series of simulations were conducted using various process parameters detailed in Table 3 and Table 4. The outcomes, including the maximum temperature, and melt pool dimensions (depth and width) were then compared against experimental data. The simulation data were compared with experiments conducted by Zhirnov et al. [51] in terms of maximum temperature and with experiments by Dilip et al. [52] regarding melt pool dimensions. Additionally, the simulated melt pool width was cross-checked with the experimental results obtained in Section 2.3. The comparison aimed to ensure the accuracy and reliability of the modeling approach and simulation input parameters. Residual stress primarily forms due to the thermal gradient and thermal volumetric changes occurring during the process. As a result, melt pool dimensions and thermal gradient are the main controlling factors in the evolution of stress. Therefore, thermal validation ensures that the presented model is also reliable in predicting thermal stress. By accurately simulating the thermal aspects of the process, the model provides reliable predictions for the resulting thermal stresses. Figure 3 presents a detailed illustration of the melt pool depth and width, as measured by the simulation models. The laser power and velocity values shown in Figure 3 are selected as an example for comparison purposes, illustrating typical melt pool behavior under these conditions. These values are not intended to represent a comprehensive range of process parameters but serve as a demonstration to assess the model’s accuracy with experimental findings.

#### 3.1.1. Maximum Temperature Validation

The simulations were carried out with process parameters reported in Table 3, similar to the study by Zhirnov et al. [51], in order to compare the results and verify the validity of the maximum temperature obtained from the presented model.

**Figure 4:**
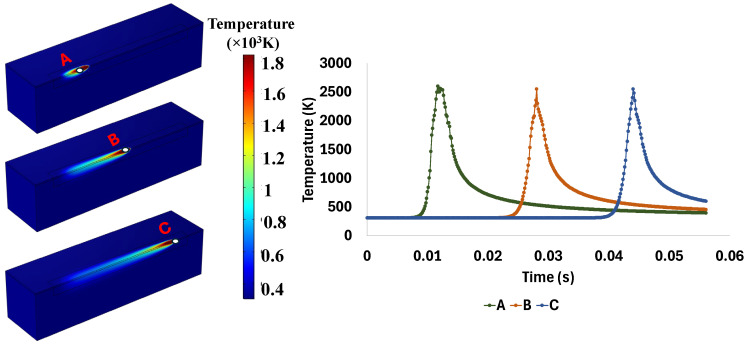
Thermal gradient at points A, B, and C, respectively, during the process with a laser power of 30 W and a laser velocity of 50 mm/s.

Figure 4 shows the thermal gradient, temperature distribution, and melt pool formation during the process in points A, B, and C along the laser scanning with a laser velocity of 50 mm/s. As the laser scans the material, the temperature begins to increase. Once it surpasses the melting point ($T_{m}$), a phase change initiates in the area experiencing the temperature over the $T_{m}$. This process results in the formation of the melt pool. For example, the temperature distribution graphs for the three selected points (A, B, and C), along with the single-track laser scan, demonstrate that when the laser passes over these points, they reach the maximum temperature, which is approximately 2500 K for all of them. The temperature decreases as the laser moves away from these selected points. The rate of temperature increase is slightly higher than the rate of temperature decrease.

**Figure 5:**
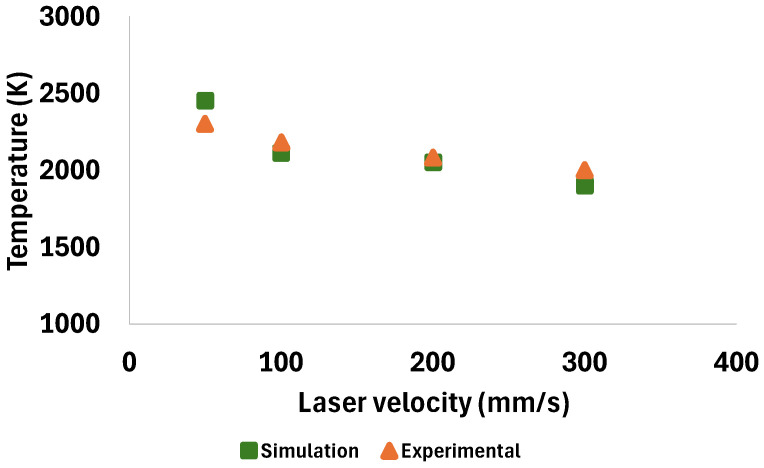
Temperature validation for the simulation model with the experimental data [51].

The reported results for the maximum temperature achieved during the process, as obtained from both the simulation and experimental data, are shown in Figure 5. It is evident that the results from both methods fall within a similar range and exhibit an acceptable trend. As shown in Figure 5, both the simulation and experimental results demonstrate a gradual decrease in the maximum recorded temperature as the laser scanning velocity increases. The maximum temperature from the simulation data aligns with the experimentally recorded maximum temperatures, with a maximum error of +6.5% for the velocity of 50 mm/s and a minimum error of +1.3% for 200 mm/s.

#### 3.1.2. Melt Pool Dimension Validation

The melt pool dimensions, including both depth and width, obtained in the current simulation were compared with the results reported by Dilip et al. [52]. Table 4 presents the process parameters used for model validation, comparing the melt pool dimensions from simulations with those from experiments.

**Figure 6:**
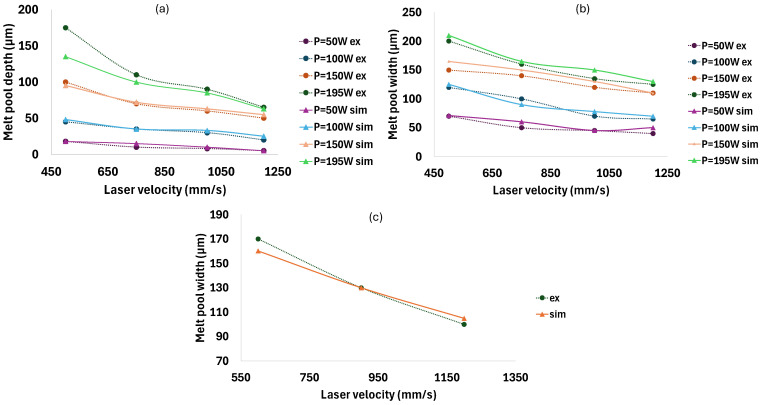
Model validation by comparing simulation and experimental results: (**a**,**b**) melt pool depth and width from simulations and experimental data [52]; (**c**) melt pool width from simulations and experiments conducted in this study (Section 2.3).

Figure 6a,b show the comparison between the obtained simulated data and experimental results from Dilip et al.’s work [52]. The simulation outcomes demonstrate a satisfactory correlation with the experimental results, reflecting a reasonable and consistent trend. As the laser power increased from 50 W to 195 W, the melt pool dimensions expanded correspondingly. This indicates that higher laser power results in deeper and wider melt pools due to the increased thermal energy supplied to the material. Conversely, when the laser scanning velocity increased from 500 mm/s to 1200 mm/s, the melt pool dimensions decreased significantly. This reduction is attributed to the fact that higher scanning velocities reduce the time for heat input in a given area, resulting in lower heat input and subsequently, smaller melt pool sizes. Overall, the model accurately captures the influence of both the laser power and velocity on melt pool size, aligning with the trends observed in the experiments. Additionally, Figure 6c illustrates the verification of the simulation model using experimental results obtained from tests conducted in Section 2.3. These results also indicate good agreement between the simulation model and experimental data, confirming the reliability and validity of the presented model.

#### 3.1.3. Residual Stress Evolution

**Figure 7:**
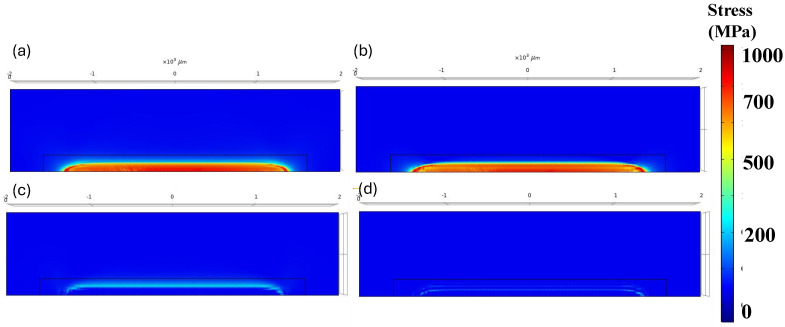
Distribution of thermal residual stress of (**a**) von Mises stress; (**b**) stress along the x-axis; (**c**) stress along the y-axis; and (**d**) stress along the z-axis.

**Figure 8:**
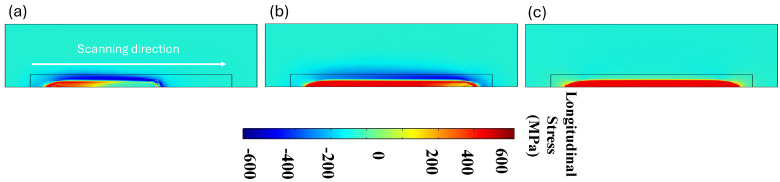
Longitudinal stress generation (**a**) during laser scanning; (**b**) immediately after scanning; and (**c**) after cooling down to room temperature.

According to the inherent characteristics of the LPBF process, which involve high thermal gradients and rapid cycles of melting and solidification, the generation of residual stress is unavoidable. To analyze residual stress formation, following model validation based on maximum temperature and melt pool size, a single-track simulation was performed using a laser power of 150 W and a scanning velocity of 600 mm/s. Figure 7a–d illustrates the distribution of von Mises stress and other stress components along the x, y, and z axes after the cooling phase to room temperature. A comparison of the results in Figure 7b–d reveals that residual stress formation is more pronounced along the x-axis (the laser scanning direction) compared to the other two directions. This happens because the laser heats up the material along its path (the x-axis), causing the material to expand. As the laser moves forward, the heated area quickly starts to cool and contract, creating stress. The material is constrained by the surrounding cooler material, so it cannot freely expand or contract. This results in higher stress in the x-direction, which is the main direction influenced by the laser’s movement. On the other hand, In the y and z directions, the material is less affected by the rapid heating and cooling caused by the laser. These directions are not directly under the laser beam during scanning, so they experience less thermal expansion and contraction compared to the x-axis. As a result, the residual stress along these axes is lower. Figure 8 presents the formation of longitudinal stress (along the x-axis) in three distinct stages: during laser scanning, immediately after laser scanning, and after complete cooling to room temperature. The results in Figure 8 clearly show the development of both tensile and compressive stresses during these stages. Based on Figure 8a,b, compressive stresses in the surrounding area of the melt pool are generated due to the thermal expansion of the heated zone. As this zone expands, cooler and stiffer material further away, which is unaffected by heat, applies opposing compressive forces to constrain the expansion, thereby creating compressive stresses in the surrounding area. Conversely, as the molten pool begins to solidify, the surrounding solid material counteracts the contraction caused by solidification by applying tensile forces. This interaction results in tensile stress within the laser scanning area and compressive stress in the surrounding region. Additionally, a small amount of tensile stress appears in areas further away, balancing out the compressive stress. Figure 8c shows that, after the cooling stage, the compressive stress decreased while the tensile residual stress along the laser scanning direction increased.

**Figure 9:**
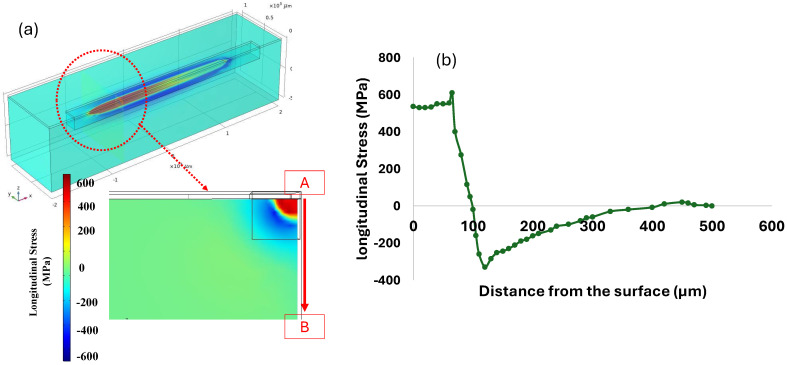
Longitudinal stress distribution from point A to B, showing the direction of stress interpretation with an arrow in (**a**) schematic view of defined sections, and (**b**) longitudinal stress profile.

The amount of generated residual stress in the x direction can be seen in Figure 9. This change in stress distribution highlights the dynamic nature of stress evolution during the melting and solidification processes, ultimately leading to the residual stresses observed in the final structure. As shown in Figure 9a, residual stress along the x-axis is not confined to the surface but extends into the material depth between points A and B. Figure 9b illustrates the longitudinal residual stress distribution as a function of distance from the surface. At the surface (Point A = 0 µm), a high tensile stress of approximately 600 MPa is observed, likely due to thermal contraction after cooling. This tensile stress remains elevated until around 100 µm, where it transitions sharply to compressive stress, reaching a minimum of −300 MPa. This compressive zone beneath the surface is likely a response to the tensile stresses generated in the upper layers. Beyond 150 µm, the stress gradually returns to positive values, stabilizing near zero around 500 µm as it approaches point B, indicating a more balanced stress state at greater depths. This pattern highlights the development of tensile stresses at the surface and compressive stresses below.

### 3.2. Effect of Process Parameters

After the validation of the simulation model, the effect of important process parameters including laser velocity, laser power, hatch space, and scanning pattern on the thermal gradient and formed residual stress distribution was studied.

To study the effect of laser velocity, simulations were conducted with a constant laser power while varying the velocity at four different rates. To examine the effect of laser power, simulations were carried out with a constant velocity while varying the power levels.

In the subsequent phase, simulations were performed with both power and velocity held constant, but with different hatch spaces to better understand the impact of hatch spacing on the results. Finally, with all process parameters—velocity, power, and hatch space—kept constant, the study focused on evaluating the effect of the laser scanning pattern by altering the scanning pattern.

This systematic approach allows a comprehensive analysis of how each parameter individually and collectively influences the residual stress formation, thereby providing deeper insights into the optimization of the laser processing technique.

#### 3.2.1. Laser Velocity

The process parameters used during simulation to study the effect of laser velocity on melt pool evolution and residual stress formation are summarized in Table 5.

**Figure 10:**
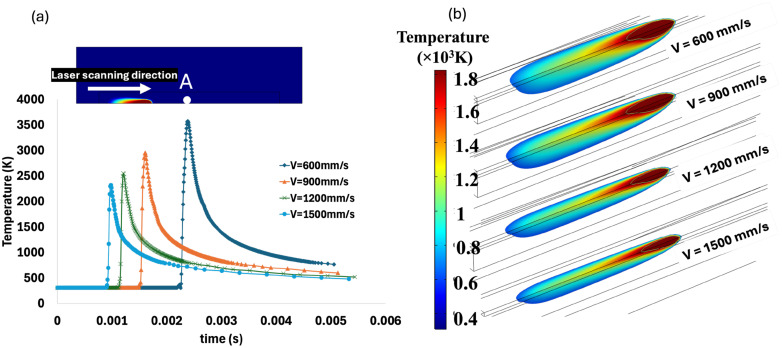
The effect of laser velocity on (**a**) thermal gradient at point A during the laser scanning process and (**b**) melt pool shape and size.

The effect of laser velocity on the thermal gradient distribution, recorded from point A, is illustrated in Figure 10a, while the evolution of melt pool size is shown in Figure 10b. As previously noted, increasing laser velocity directly reduces both the heat input and the duration of energy absorption. Consequently, as the laser moves faster across the material, less thermal energy is transferred into the powder bed, leading to lower peak temperatures and a reduced volume of molten material. The diagram of the thermal gradient in Figure 10a shows a wider distribution with a laser velocity of 600 mm/s, which reflects the longer interaction time between the laser and the material becomes narrower as the velocity increases. The narrowest gradient with the lowest reported maximum temperature occurs at the laser velocity of 1500 mm/s (Figure 10a). This is because the faster the laser scans, the less time the material must absorb heat, causing the thermal energy to be concentrated in a smaller area.

Figure 10b further demonstrates the relationship between laser velocity and melt pool size. At 1500 mm/s, the melt pool dimensions are noticeably smaller compared to those at 600 mm/s. This reduction in melt pool size is correlated with the increased scanning velocity, which constrains the quantity of heat absorbed by the material. As the laser scans the surface more rapidly, the duration of interaction between the laser and the material diminishes, resulting in a decreased transfer of thermal energy to the powder bed. Consequently, the volume of material attaining the melting point decreases, leading to a smaller molten region. In general, the melted area decreases as the laser velocity increases. As shown in Figure 10b, the laser velocity also influenced the shape of the melt pool. As laser scanning velocity increased, the melt pool became more elongated. On the other hand, at lower scanning velocity, the shape of the melt pool was noticeably broader and more circular.

**Figure 11:**
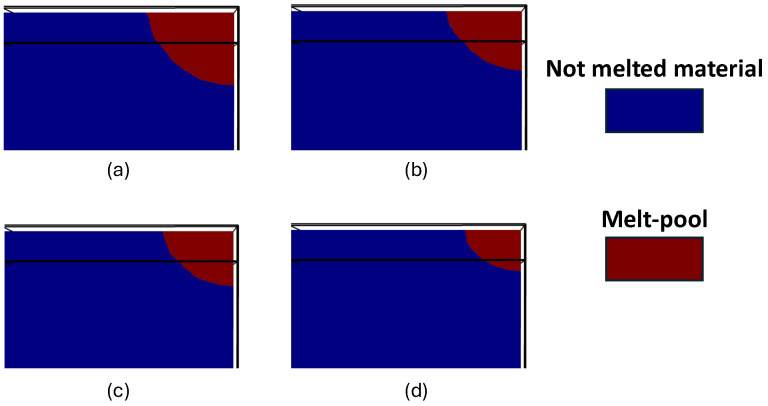
Effect of laser velocity on melt pool dimensions, under velocity of (**a**) 600 mm/s; (**b**) 900 mm/s; (**c**) 1200 mm/s; and (**d**) 1500 mm/s.

The cross-section of the melt pools, presented in Figure 11, shows that variations in laser velocity affect both the depth and width of the melt pool. By increasing the laser velocity, the depth of penetration decreased due to the reduced absorption time. The width of the melt pool was also smaller at higher velocities. Keshavarzian et al. also concluded similar results in an experimental study for Hastalloy X [55].

Achieving full penetration along the entire scanned line is a critical factor in determining the mechanical properties of the LPBF process. Higher scanning velocities can increase the likelihood of porosity and compromise full penetration [56]. As a result, at faster laser velocities, the risk of incomplete penetration and lack of fusion rises, potentially jeopardizing the integrity of the final part.

Having addressed the influence of laser velocity on the thermal gradient and melt pool dimensions, it is crucial to explore how this factor affects residual stress distribution within the material specifically along the laser scanning direction. The variations in temperature and melt pool shape associated with different laser velocities potentially contribute to the development of residual stresses during the LPBF process.

**Figure 12:**
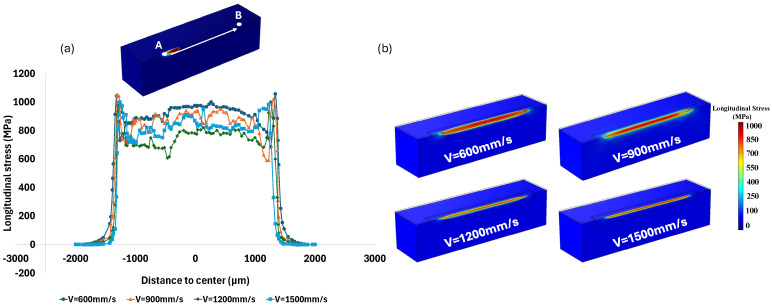
(**a**) Diagram of longitudinal stress gradient along the laser scanning; and (**b**) contour plot of longitudinal stress distribution under various laser scanning velocities.

Figure 12a shows the effect of laser velocity on the distribution of longitudinal residual stress along the laser scanning direction after cooling to room temperature, from point A to point B which are 4000 μm apart. At a velocity of 600 mm/s, the average longitudinal stress from point A to B along the laser scanning was reported to be approximately 1000 MPa, while it was approximately 900 MPa for 900 mm/s, 700 MPa for 1200 mm/s, and 800 MPa for 1500 mm/s. Notably, the magnitude of thermal stress initially decreased with increasing laser velocity from 600 mm/s to 1200 mm/s. As previously mentioned, increasing laser velocity results in a smaller melt pool, reducing the area undergoing phase change. This smaller area experiences less contraction and expansion, resulting in a smaller area with thermal stress and thus less stress as laser velocity increases. However, increasing laser velocity also causes a higher cooling rate as the heat source moves away more rapidly, and the thermal gradient becomes steeper due to rapid heating and cooling. The magnitude of thermal stress began to increase as the velocity increased from 1200 mm/s to 1500 mm/s. Hence, as the velocity increases, longitudinal stress initially decreases and subsequently increases. This trend was also reported by Liu et al. in an experimental study for 316L-Stainless Steel [57]. Figure 12b presents a contour plot of residual stress along the laser scanning direction at various laser velocities, with the red areas indicating regions of higher tensile residual stress formed after cooling.

**Figure 13:**
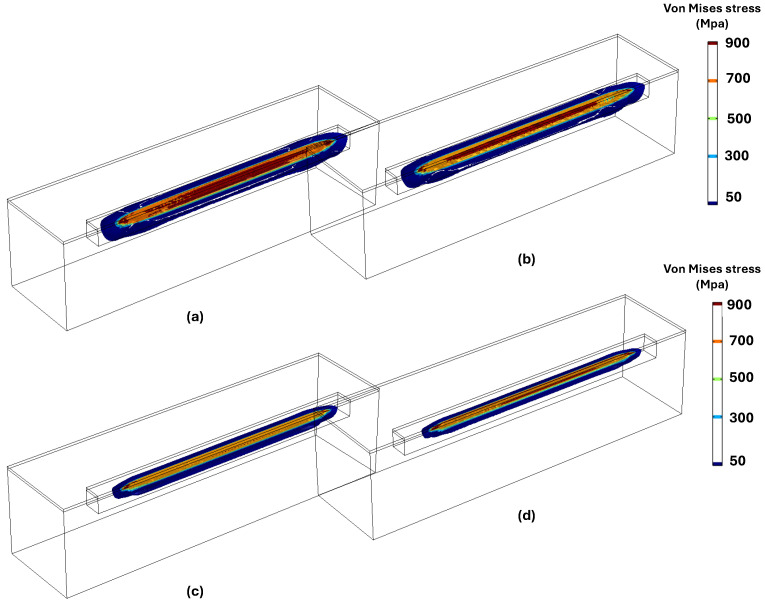
Von Mises stress distribution under velocity of (**a**) 600 mm/s; (**b**) 900 mm/s; (**c**) 1200 mm/s; and (**d**) 1500 mm/s.

Figure 13 illustrates the distribution of von Mises stress at different laser velocities of 600, 900, 1200, and 1500 mm/s. At lower velocities of 600 mm/s and 900 mm/s, the von Mises stress is higher, and the area where the stress exceeds 900 MPa is more extensive. The stress spreads across a wider region, and stress concentration is more pronounced at these lower velocities (600 mm/s and 900 mm/s) compared to the higher velocities (1200 mm/s and 1500 mm/s). Furthermore, the locations of stress concentration shift as the laser velocity increases. At lower velocities, stress concentration is primarily located along the center of the laser scanning path, whereas at higher velocities, the stress tends to concentrate around the periphery and near the edges of the scanning line. The trend of stress variation with laser velocity follows a similar pattern to that observed for longitudinal stress. As the laser velocity increases up to 1200 mm/s, the von Mises stress initially decreases but then shows a slight increase beyond this point as the velocity increases from 1200 mm/s to 1500 mm/s.

It is important to note that while increasing laser velocity can generally decrease the thermal residual stress (until the critical point) and control stress concentrations and distortions, it also increases the risk of lack of fusion and porosity formation during the process [56,58,59,60]. Therefore, selecting the appropriate velocity as a process input parameter is a critical consideration that should be addressed by evaluating both thermal and mechanical effects.

#### 3.2.2. Laser Power

To study the effect of laser power on temperature distribution, melt pool dimension, and thermal residual stress, several simulations were conducted. The simulation process parameters are chosen according to Table 6, with constant velocity and varied laser power.

**Figure 14:**
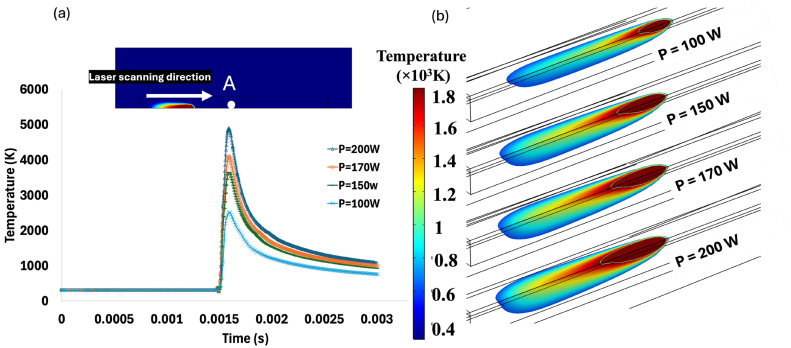
Effect of laser power on: (**a**) thermal gradient diagram; and (**b**) melt pool volume.

As anticipated, the results in Figure 14a demonstrate that increasing the laser power from 100 to 200 W leads to a significant rise in the maximum temperature at point A during the process from approximately 2000 K at 100 W to 5000 K at 200 W. The higher laser power introduces more energy into the material at the constant scanning velocity, leading to elevated temperatures, steeper thermal gradients, and an enlarged melt pool zone. Figure 14b illustrates the shape and volume of the melt pool at different laser power levels, showing that the melt pool volume increases as the laser power rises.

**Figure 15:**
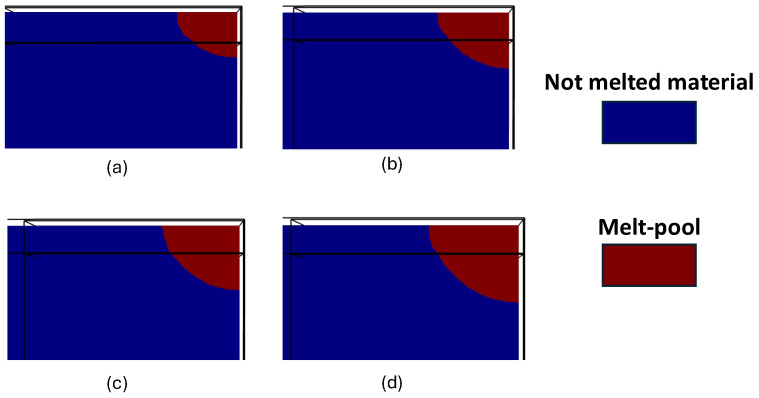
Effect of laser power on melt pool dimensions under power of (**a**) 100 W; (**b**) 150 W; (**c**) 170 W; and (**d**) 200 W.

Figure 15 indicates that both the depth and width of the melt pool increase with higher laser power. As the laser power increases, a greater amount of energy is delivered to the material, which enhances the heat input and extends the melt pool in terms of both penetration depth and lateral expansion, as stated with an experimental and numerical analysis by Pramod and Kesavan [61]. This increased energy absorption facilitates deeper melting and a broader molten zone, thereby contributing to an enlarged melt pool overall. The deepest melt pool was observed at the highest laser power of 200 W, while the shallowest melt pool depth was seen at the lowest power of 100 W. This direct relationship between laser power and melt pool depth highlights the significant impact of increased energy input on the melting behavior of the material. In this case, similar to the effect of laser velocity, higher laser power could enhance the likelihood of full penetration, and on the other hand, reduce the risk of lack of fusion throughout the scanned line which was also confirmed experimentally [60].

**Figure 16:**
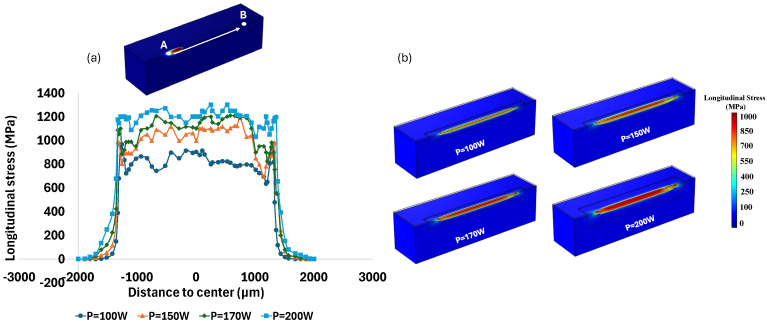
(**a**) Diagram of longitudinal stress gradient along the laser scanning; and (**b**) contour plot of longitudinal stress distribution under various laser powers.

**Figure 17:**
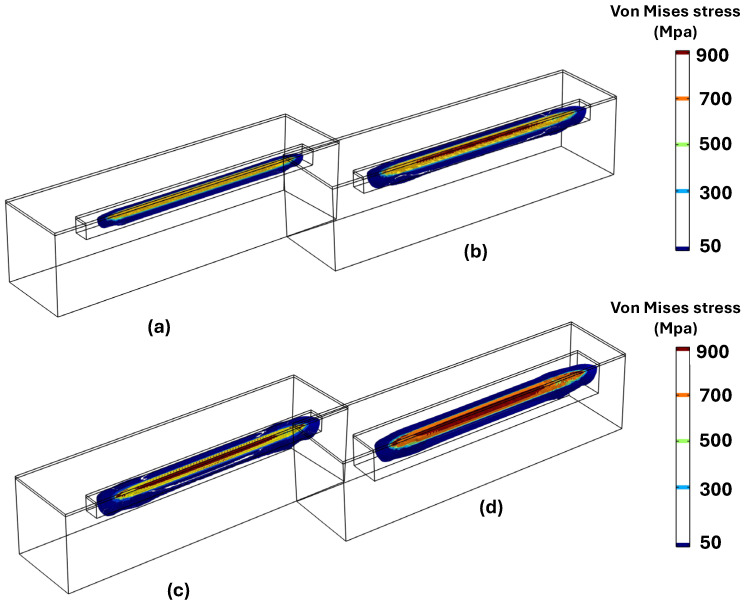
Von Mises stress distribution under power of (**a**) 100 W; (**b**) 150 W; (**c**) 170 W and (**d**) 200 W.

As shown in Figure 16a, laser power variation also affected formed thermal residual stress after cooling to room temperature, from point A to point B, which are 4000 μm apart. This is in addition to the effect of laser power value on the melt pool volume, as explained above and shown in Figure 15. According to Figure 16a, the average longitudinal stress along the laser scanning direction is approximately 568 MPa at a laser power of 100 W. This value increases to 727 MPa when the power rises to 150 W. A slight increase in power from 150 W to 170 W results in a marginal rise in the average longitudinal stress to 780 MPa. The highest average longitudinal stress, 902 MPa, occurs at the maximum power of 200 W. The magnitude of thermal stress, both longitudinal (Figure 16b) and von Mises (Figure 17), increases significantly when the power increases from 100 W to 200 W. The area of stress distribution expands with increasing laser power. The rise in melt pool volume, driven by the increased laser power and corresponding heat input, results in a larger area undergoing phase change. The melting and solidification processes, along with the associated expansion and contraction, generate thermal stress along the laser scanning path. As a result, a larger melted area leads to higher thermal residual stress. This expanded area under higher residual stress is clearly shown in Figure 16b and Figure 17. Similarly, the area affected by von Mises stress distribution widens as the power increases from 100 W to 200 W, as depicted in Figure 17. Additionally, the maximum stress concentration is higher at 200 W compared to lower power levels. These results were also concluded in a similar study by Mukherjee et al. [62]. While higher laser power leads to higher residual stress, it also reduces the possibility of porosity formation and incomplete penetration defects. Therefore, both aspects should be considered when selecting the appropriate laser power for the process.

#### 3.2.3. Hatch Space

To study the effect of hatch space on the thermal gradient and resulting thermal residual stresses, both laser power and velocity were kept constant during the process. The model was repeated using five laser scanning lines with three different hatch space values. At this step, the simulation process parameters were selected based on the data in Table 7.

**Figure 18:**
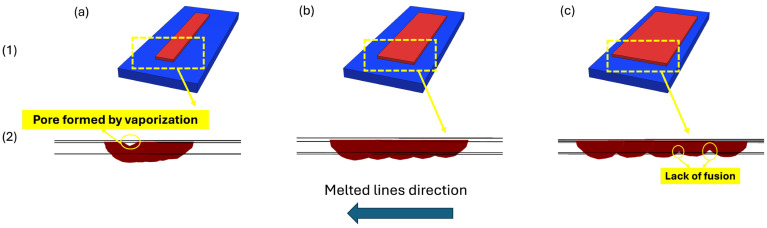
The effect of hatch space values of (**a**) 25 μm; (**b**) 50 μm; and (**c**) 75 μm, on (**1**) the schematic representation of the deposited width and (**2**) the cross-sectional view of the melted regions.

Figure 18 presents a schematic of multi-track laser scanning along with the cross-sectional view of the melted lines, obtained from simulations for three different hatch space values. It highlights the effect of hatch space on scanned area dimensions, shown schematically (Figure 18(a1–c1)), as well as the melt pool size, illustrated in the cross-sectional view (Figure 18(a2–c2)). In all cases, the size of the melt pool increases with each successive track. For instance, in Figure 18(c2), following the direction of the melted lines, the melt pool in track 2 is larger than in track 1 and track 5 has the largest melt pool. This is because each preceding track preheats the material for the next track, causing the process to begin at a higher initial temperature. Under constant heat input, this leads to an increase in melt pool size. As a result, each successive track produces a larger melt pool than the previous one.

As shown in the schematic overview of melted lines in Figure 18(1), decreasing the distance between the lines (lower hatch space) has a notable impact on the width of the area scanned by the laser. As the hatch space reduces, the laser scans a more confined region, which in turn, reduces the overall width of the scanned area. This reduction in scanned area directly influences the deposition rate, leading to a slower production rate. At a hatch space of 25 µm, the width of the deposited area is 35% and 50% smaller, compared to the wider hatch spaces of 50 µm and 75 µm, respectively.

However, despite the larger scanned area at a 75 µm hatch space, Figure 18(c2) shows evidence of a lack of fusion in the cross-sectional view of the melt pool when the hatch space is set to 75 µm. This issue was resolved by reducing the hatch space to 50 µm and 25 µm, where no un-melted areas were observed between the lines. With smaller hatch space values, the lines are closer together, increasing the likelihood of full penetration and defect-free deposition. In contrast, larger hatch space increases the risk of lack of fusion and porosity formation because the melt pools are smaller (due to less preheating by successive tracks) and spaced farther apart. Increasing the overlap between lines, and consequently between the melt pools, ensures a dense, void-free deposition and reduces the risk of lack of fusion.

Therefore, while a smaller hatch space improves the chance of full penetration and reduces the risk of defects such as lack of fusion or porosity in the deposited lines, it results in a lower deposition rate. Furthermore, reducing the hatch space increases heat accumulation and consequently, elevates the maximum temperature experienced by any point of material [63,64]. It can be hypothesized that, at a 25 µm hatch space, the maximum temperature experienced by the material exceeded the vaporization temperature, causing part of the melted material to evaporate during heating. This evaporation led to the formation of pores and porosities on the surface of the deposited area after solidification, as shown in Figure 18(a2).

**Figure 19:**
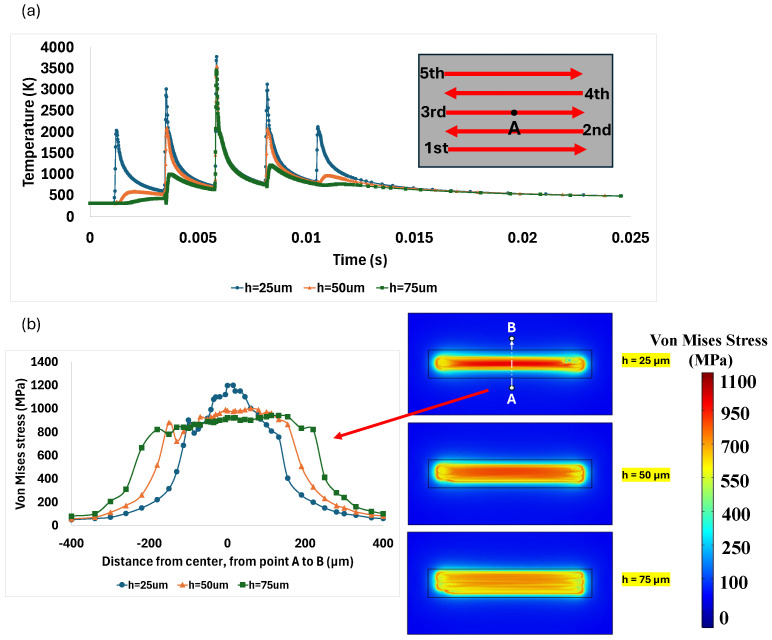
(**a**) Temperature change diagram at point A for different hatch spaces during laser scanning process; (**b**) effect of hatch space on residual stress.

As the hatch space impacts both the thermal gradient and melt pool dimensions, it is expected to influence thermal residual stresses as well. Figure 19a demonstrates the connection between the hatch space values and the thermal gradient at point A. Increasing the hatch space from 25 µm to 75 µm reduced the thermal gradient at this point due to the lower heat concentration. At a 25 µm hatch space, point A melted in each laser scanning track since the maximum temperature exceeded the material melting point. The smaller distance between the lines, with constant power and velocity, increases the maximum temperature, causes heat accumulation, and results in a higher thermal gradient, as stated by Nazami et al. [65]. When the hatch space increased to 50 µm, point A slightly melted in track 2 and then remelted in tracks 3 and 4, according to the temperature diagram presented in Figure 19a. The results indicate that as the hatch space increases, the reduced proximity between the lines lowers the thermal gradient, which in turn decreases the cooling rate. Moreover, the heating and cooling cycles become milder at larger hatch spaces. Equation (18) shows the volumetric energy density input into the material at each line.

(18) $$E_{V}=\frac{P}{v\times h\times d}$$

where $E_{V}$ is volumetric energy density, P is laser power, v is laser velocity, h is hatch space, and d is the powder layer thickness. According to Equation (18), the volumetric energy density decreases as hatch space increases due to their inverse relationship. In other words, increasing the hatch space reduces the energy density, leading to a decrease in the total accumulated energy, as indicated by the lower maximum temperature shown in Figure 19a. This also decreases the number of heating and cooling cycles. As a result, the overlap between scanning lines is minimized, and the formed thermal residual stress is reduced. These findings have been experimentally confirmed by Ji et al. and Mansouri et al. [63,64]. The simulation results confirmed these observations, showing lower residual stress at the largest hatch space. Figure 19b illustrates the residual stress distribution between points A and B, along with the contour maps of residual stress for each hatch space after cooling. The lowest residual stress occurred at the largest hatch space of 75 µm. As the hatch space was reduced from 50 µm to 25 µm, the maximum residual stress increased by 20%. Specifically, decreasing the hatch space from 75 µm to 25 µm resulted in an increase in maximum thermal residual stress of 30%. Thus, the hatch space exhibited an inverse relationship with thermal residual stress. While a larger hatch space decreases heat accumulation and reduces the maximum residual stress, a smaller hatch space improves the chances of full penetration and minimizes the risk of defects, such as lack of fusion or porosity in the deposited lines. Additionally, as it was mentioned before, a smaller hatch space can decrease the production rate. Therefore, it is crucial to note that there is an optimal hatch space that ensures sound deposition, controls residual stress, and maintains production efficiency.

#### 3.2.4. Laser Scanning Pattern

**Figure 20:**
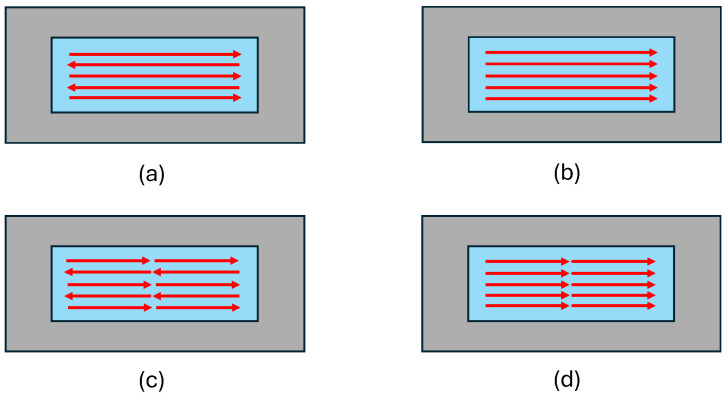
Schematic of laser scanning patterns: (**a**) alternating; (**b**) unidirectional; (**c**) 2 domains, alternating; and (**d**) 2 domains, unidirectional.

To investigate the effect of laser scanning patterns on thermal distribution and thermal residual stress formation, the process was simulated using a constant laser velocity of 1200 mm/s, a constant laser power of 150 W, a hatch space of 50 µm, a laser spot size of 100 µm, and four different laser scanning patterns as shown in Figure 20.

**Figure 21:**
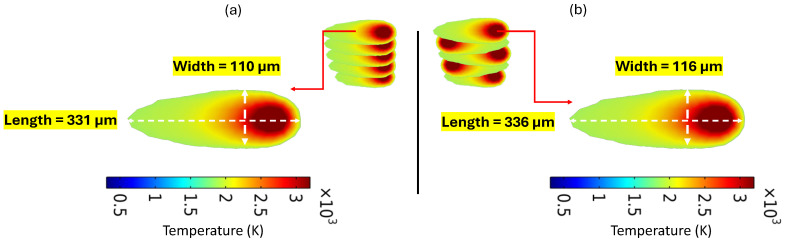
Melt pool dimension obtained from simulation model for (**a**) unidirectional and (**b**) alternating scan patterns.

According to Figure 21, the scanning pattern had a negligible effect on the melt pool dimensions. Whether using unidirectional or alternating scan strategies, the size and shape of the melt pool remained largely consistent under the given conditions. However, in the alternating scanning pattern, the melt pool was slightly larger. It was observed that the melt pool dimensions in both alternating and unidirectional scanning patterns were identical to those in the two alternating domains and the two domains with unidirectional patterns. Therefore, Figure 21 is used as a representative for both initial patterns.

**Figure 22:**
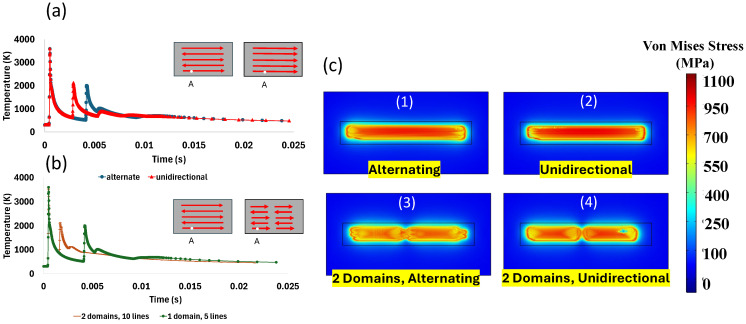
Thermal gradient for comparing (**a**) alternate and unidirectional and (**b**) one domain and two domains scanning pattern, and (**c**) residual stress distribution under different scan patterns, (**c1**) alternating, (**c2**) unidirectional, (**c3**) alternating, 2 domains, and (**c4**) unidirectional, 2 domains.

Figure 22 shows the effect of the laser scanning pattern on the thermal gradient at point A (the beginning of track 1) and thermal residual stress after the cooling stage. When the scanning pattern is unidirectional, the laser only moves in one direction and all the scanning lines are in the same direction. The diagram of the thermal gradient reported at point A shows slightly sharper variations according to simulation results, and consequently, residual stress is higher compared to alternating scanning patterns. In the alternating scan strategy, each new scan starts in a region that is already at a higher temperature because the previous scan finished nearby. This higher starting temperature in the alternating scan lowers the temperature difference, or gradient, compared to unidirectional scanning. As a result, the temperature change at the beginning of each scan is less in the alternating pattern than in the unidirectional one, as seen in Figure 22a. Although the distribution and the amount of residual stress for both scanning patterns are nearly identical as it gets close to the middle of the scanned region, the residual stress is higher at the beginning and end of each track in the unidirectional scan pattern (Figure 22(c1,c2)).

In the next step, the scanning pattern was changed again, replacing 5 long lines with 10 shorter lines in two domains. As the length of the lines was reduced to half, the thermal gradient at point A also decreased due to a more uniform temperature distribution (Figure 22b). Consequently, the amount of residual stress also decreased with the scanning patterns of the two domains. Similarly, less residual stress was observed with the alternative domain pattern (Figure 22(c3,c4)).

#### 3.2.5. Overall Residual Stress Results Under Studied Process Parameters

**Figure 23:**
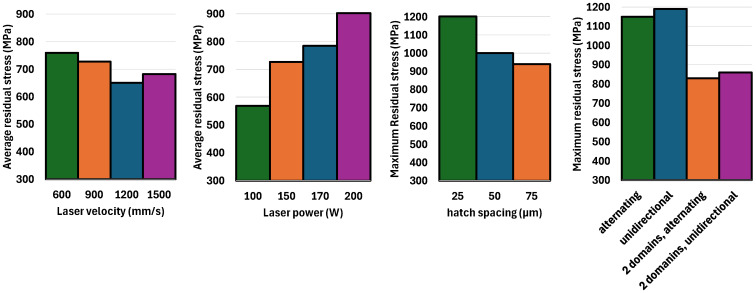
The effect of different process parameters on thermal residual stress.

To provide a clearer comparison of the effects of the various process parameters studied in this research, including laser velocity, laser power, hatch space, and scanning pattern, Figure 23 presents a summary in the form of a bar chart. Among these factors, laser power and shortening the scanning line length had the most significant effect on residual stress. Increasing laser power from 100 W to 200 W led to a 60% rise in residual stress, increasing it from 568 MPa to 902 MPa. In contrast, doubling the laser velocity resulted in only a 15% reduction in residual stress. The maximum residual stress for hatch spaces of 25 µm, 50 µm, and 75 µm was reported to be 1200 MPa, 1000 MPa, and 940 MPa, respectively. Switching the scanning pattern from alternating to unidirectional had minimal impact on residual stress; however, when the pattern shifted to two domains with shorter scan lines, residual stress dropped considerably from 1100 MPa to 800 MPa, which was about a 30% reduction. As shown in Figure 23, laser power had the greatest effect on residual stress, while other process parameters had a comparatively lower impact.

## 4. Conclusions

A FEM was developed to investigate heat transfer and residual stress formation during single-track and multi-track laser scanning of Ti-6Al-4V alloy in the LPBF process. Key insights into thermal gradients, melt pool dynamics, and residual stress distribution were obtained. The main findings are as follows:

- A volumetric heat source with a decaying absorptivity function, accounting for laser velocity and power, provided results that aligned with experimental data;
- Thermal cycles of heating and cooling in each track caused material melting and solidification. The material melted when temperatures exceeded the melting point and solidified as the laser moved away, cooling to an ambitious temperature;
- There were also three stages of thermal stress formation. Stress initially appeared in the first cycle of heating, then decreased by melting, and again increased significantly by solidification. Tensile stress increased with cooling, while compressive stress decreased;
- Thermal cycles and phase change are the main controlling factors in the formation of residual stress. Increasing the thermal gradient and greater shrinkage volume results in higher residual stress;
- Increasing laser velocity from 600 mm/s to 1500 mm/s reduced melt pool size, initially decreasing residual stress at 1200 mm/s and slightly increasing it at 1500 mm/s due to higher cooling rates;
- Higher laser power increased the thermal gradient, melted area, and residual stress;
- Increasing hatch space from 25 µm to 75 µm led to a wider deposited area but introduced a lack of fusion at 75 µm, while 25 µm caused surface pores due to material vaporization;
- Hatch space affected the melted area and residual stress distribution. At 75 µm, wider areas melted, with reduced residual stress due to lower heat accumulation and volumetric heat density;
- Unidirectional scanning resulted in sharper thermal gradients and higher residual stress while alternating and domain patterns produced more uniform heat distribution and lower residual stress.

## Figures and Tables

**Table 1:** Thermal properties for Ti-6Al-4V used in the simulation [43,45,46].

Properties	Values
Melting point, *T_m_* (K)	1923
Vaporization point, *T_v_* (K)	3660
Latent heat, L (kJ/kg)	286
Density, *ρ* (kg/m^3^)	4512 − 0.154 T 308 < T < 1923
Thermal conductivity, *k* (W/m·K)	1.25 + 0.015 T 308 < T < 1268 3.15 + 0.012 T 1268 < T < 1923
Specific heat, *C_p_* (J/kg·K)	483.04 + 0.215 T 308 < T < 1268 412.7 + 0.180 T 1268 < T < 1923

**Table 2:** Mechanical properties of Ti-6Al-4V used in the simulation model [12,18,36].

Temperature (K)	Young’s Modulus (GPa)	Poisson’s Ratio	Yield Strength (MPa)	Coefficient of Thermal Expansion (×10^−6^)
293	125	0.34	1000	8.78
478	100	0.36	663	10
701	80	0.38	500	11.1
923	55	0.42	300	11.7
1145	20	0.43	25	12.3
1367	5	0.43	5	12.4
1923	0.3	0.43	0.3	12.5

**Table 3:** Process parameters used in simulation to validate the simulation model.

Process Parameters	Value
Laser power (W)	30
Laser velocity (mm/s)	50, 100, 200, 300
Laser spot diameter (µm)	70

**Table 4:** Simulation parameters used for validating the simulation model.

Process Parameters	Value
Laser power (W)	50, 100, 150, 170, 195
Laser velocity (mm/s)	500, 600, 750, 900, 1200
Laser spot diameter (µm)	100

**Table 5:** Simulation process parameters used to study the effect of laser velocity.

Process Parameters	Values
Laser power (W)	150
Laser velocity (mm/s)	600, 900, 1200, 1500
Laser spot diameter (µm)	100

**Table 6:** Simulation process parameters to study the effect of laser power.

Process Parameters	Values
Laser power (W)	100, 150, 170, 200
Laser velocity (mm/s)	900
Laser spot diameter (µm)	100

**Table 7:** Simulation process parameters to study the effect of hatch space.

Process Parameters	Value
Laser power (W)	150
Laser velocity (mm/s)	1200
Hatch space (µm)	25, 50, 75
Laser spot diameter (µm)	100

## Data Availability

The original contributions presented in the study are included in the article. Further inquiries can be directed to the corresponding author.
